# Mendelian randomization study indicates lack of causal associations between iron status and lung cancer

**DOI:** 10.1097/MD.0000000000029879

**Published:** 2022-07-22

**Authors:** Hong Qin, Weibiao Zeng, Yongfu Lou

**Affiliations:** a Department of Respiratory, People’s Hospital of Shangrao City, 76 Shuyuan Road, Shangrao, P. R. China; b Department of Thoracic Surgery, The First Affiliated Hospital of Soochow University, Suzhou, P. R. China; c Department of Thoracic Surgery, People’s Hospital of Shangrao City, 76 Shuyuan Road, Shangrao, P. R. China.

**Keywords:** causal relationship, iron status, lung cancer, Mendelian randomization

## Abstract

Observational studies provided conflicting results on the association between iron status and the risk of lung cancer. The aim of our study was to investigate the effect of genetically determined iron status on lung cancer risk using a mendelian randomization (MR) approach.

Single-nucleotide polymorphisms for iron status were selected from a genome-wide meta-analysis of 48,972 subjects. Genetic association estimates for risk of lung cancer were derived from a Genome-Wide Association Study (GWAS) summary performed by the International Lung Cancer Consortium. The inverse-variance weighted method was used for the main analyses and sensitivity analyses.

MR analysis demonstrated that increased genetically-predicted iron status did not causally increase risk of lung cancer. The odds ratios were 1.11 (95% CI, 0.92, 1.34; *P* = .26), 0.76 (95% CI, 0.52, 1.12; *P* = .17), 1.09 (95% CI, 0.86, 1.38; *P* = .47), and 0.91 (95% CI, 0.81, 1.02; *P* = .11) per 1 standard deviation increment of serum iron, ferritin, transferrin saturation, and transferrin levels, respectively. No observed indication of heterogeneity (*P* for Q > 0.05) or pleiotropy (*P* for intercept > 0.05) were found from the sensitivity analysis.

The MR study indicated that genetic iron status was not causally associated with the risk of lung cancer, the causal relationship between iron status and lung cancer needs to be further elucidated by additional studies that strictly control for confounding factors.

## 1. Introduction

According to the latest global cancer statistics, lung cancer is the second most frequent cancer worldwide and accounts for 11.4% of total cancer cases.^[1]^ Approximately 2.2 million new cases of lung cancer have been reported in 2020.^[1]^ Lung cancer is the leading cause of cancer deaths, accounting for 18.0% of all cancer deaths. Iron plays a crucial role in oxygen transport, DNA biosynthesis, and energy metabolism and is an essential nutrient for human health.^[1]^ In addition, iron induces the formation of hydroxyl radicals, which leads to oxidative damage, and thus promotes tumorigenesis.^[2]^ Some studies have found that high iron levels may be related to lung cancer risk.^[3]^ However, available epidemiological evidence examining the effect of iron status on lung cancer is inconclusive. A case-control study by Zhou et al found that iron intake was associated with an increased risk of lung cancer after combining smoking history and other potential risk factors.^[4]^ These results were consistent with findings from a study by Daniel et al that showed ferritin and other markers of iron status were significantly associated with lung cancer risk.^[5]^ The European Prospective Investigation into Cancer and Nutrition study showed that heme iron was associated with lung cancer risk and nonheme iron intake was negatively associated with lung cancer risk.^[6]^ These results from observational studies were susceptible to a variety of confounding factors, such as postnatal habits, social status, and environmental factors, all of which have the potential to influence the association between iron status and lung cancer risk.^[7]^ In addition, the possible inverse causal relationship between iron status and lung cancer risk may lead to biased results in observational epidemiological studies. Therefore, the association between iron status and lung cancer risk has been highly controversial.

Mendelian randomization (MR) analysis is an epidemiological method that uses genetic variants related to exposure as instrumental variables to assess the potential causal relationship between exposure and outcome.^[8]^ The MR approach can diminish the confounding and reverse causality since genetic information should be free of confounding factors and independent of disease state. Moreover, genetic variants follow a random distribution at the time of conception, similar to the random assignment of participants to different groups in a randomized controlled trial.^[7]^

To our knowledge, there are no MR-based studies that examine the association between iron status and lung cancer risk. In the present study, we conducted a 2-sample MR analysis using publicly available data to explore the causal relationship between 4 different iron biomarkers and lung cancer risk, including serum iron, ferritin, transferrin saturation, and transferrin.

## 2. Methods

**Figure 1.**
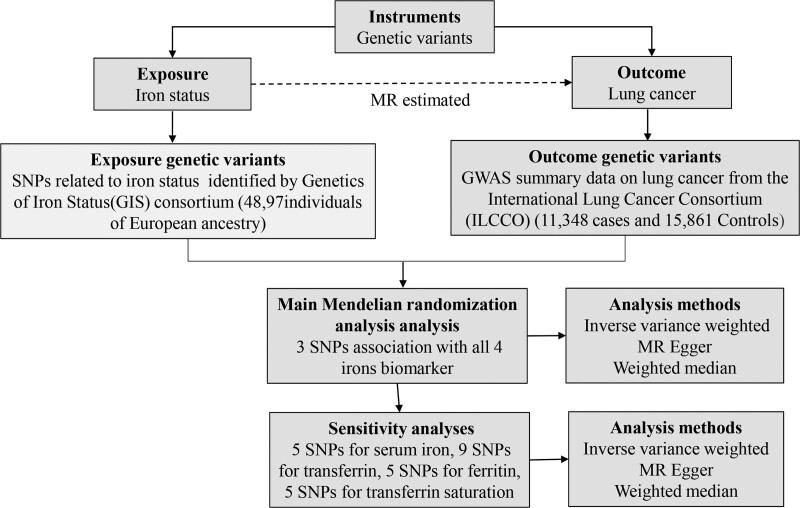
Data sources and analysis plan used in the 2-sample Mendelian randomization analysis. A summary of SNP phenotypes was obtained from publicly available GWAS databases. Three SNPs associated with all 4 iron biomarkers (increased ferritin, serum iron, transferrin saturation, and decreased transferrin) were used in the main MR analysis. SNPs affiliated with at least one of the iron markers (5 SNPs for serum iron, 9 SNPs for transferrin, 5 SNPs for ferritin, and 5 SNPs for transferrin saturation) were used in the sensitivity analysis. MR, Mendelian randomization; SNP, single nucleotide polymorphism; MR Egger, Mendelian randomization–Egger regression method.

The present 2-sample MR study used GWAS summary data obtained from the Genetics of Iron Status (GIS) Consortium^[9]^ and International Lung Cancer Consortium (ILCCO).^[10]^ These original studies were conducted with obtained ethical approval and informed consent from participants, therefore no further sanction was required. The data sources and analysis plan used in the mendelian randomization analysis is summarized in Figure 1.

### 2.1. Genetic instruments for iron status

Single-nucleotide polymorphisms (SNPs) for iron status were obtained from the GWAS summary performed by the GIS consortium,^[9]^ which combined data from 11 discovery and 8 replication cohorts encompassing 48,972 individuals of European ancestry. Increased serum iron, transferrin saturation, ferritin, and decreased transferrin were shown to be associated with elevated systemic iron status.^[11]^ SNPs directionally associated with these 4 biomarkers were considered genetic tools in the present study. Following screening, 5 SNPs were associated with serum iron, 5 SNPs with transferrin saturation, 6 SNPs with ferritin, and 8 SNPs with transferrin at the genome-wide significance threshold (*P* < 5 × 10^–8^). Three SNPs [rs1800562 and rs1799945 in hemochromatosis (HFE) and rs855791 in the transmembrane protease serine 6 gene (TMPRSS6)] out of these SNPs demonstrated an association with all 4 iron biomarkers (Table 1). Many studies have demonstrated that HFE and TMPRSS6 can regulate systemic iron status through multiple pathways (Table 2).^[12–14]^

**Table 1:** Association estimates for SNPs associated with biomarkers of iron status at genome-wide significance identified from the Genetics of Iron Status Consortium GWAS meta-analysis.

				Serum iron, μmol/L			Transferrin saturation, %			Log_10_ ferritin, mg/L			Transferrin, g/L		
SNPs	Gene	EA	EAF	Beta	SE	P	Beta	SE	P	Beta	SE	P	Beta	SE	P
rs1800562*	HFE	A	0.07	0.328	0.016	2.9 × 10^–92^	0.577	0.016	2.2 × 10^–270^	0.204	0.016	1.5 × 10^–38^	-0.479	0.016	8.9.×10^–196^
rs1799945*	HFE	G	0.15	0.189	0.01	1.1 × 10^–81^	0.231	0.01	5.1 × 10^–109^	0.065	0.01	1.7 × 10^–10^	^–^0.114	0.01	9.4 × 10^–30^
rs855791*	TMPRSS6	G	0.55	0.181	0.007	4.3 × 10^–139^	0.19	0.007	6.4 × 10^–137^	0.055	0.007	1.4 × 10^–14^	^–^0.044	0.007	2.0 × 10^–9^
rs8177240	TF	G	0.35	0.066	0.007	6.6 × 10^–20^	0.1	0.008	7.2 × 10^–38^	NA	NA	NA	0.38	0.007	8.4 × 10^–610^
rs7385804	TFR2	A	0.62	0.064	0.007	1.4 × 10^–18^	0.054	0.008	6.1 × 10^–12^	NA	NA	NA	NA	NA	NA
rs744653	AC013439.4	C	0.16	NA	NA	NA	NA	NA	NA	0.089	0.01	8.4 × 10^–19^	NA	NA	NA
rs411988	TEX14	G	0.44	NA	NA	NA	NA	NA	NA	0.044	0.007	1.6 × 10^–10^	NA	NA	NA
rs651007	ABO	C	0.79	NA	NA	NA	NA	NA	NA	0.05	0.009	1.3 × 10^–8^	NA	NA	NA
rs4921915	NAT2	A	0.76	NA	NA	NA	NA	NA	NA	NA	NA	NA	0.079	0.009	7.1 × 10^–19^
rs174577	FADS2	A	0.36	NA	NA	NA	NA	NA	NA	NA	NA	NA	0.062	0.007	2.3 × 10^–17^
rs9990333	TFRC	C	0.53	NA	NA	NA	NA	NA	NA	NA	NA	NA	0.051	0.007	2.0 × 10^–13^
rs6486121	ARNTL	C	0.34	NA	NA	NA	NA	NA	NA	NA	NA	NA	0.046	0.007	3.9 × 10^–10^

EA = effect allele, EAF = effect allele frequency, NA = not applicable, SE = standard error, SNP = single nucleotide polymorphism.

* SNPs used in the main MR analyses.

**Table 2:** Biological effects of genes corresponding to used SNPs on systemic iron status.

SNPs	Corresponding gene	Link to iron status	References (PMID)
rs1800562	HFE	HFE can regulate iron uptake by competitively inhibiting the TRF1 transferrin receptor.^[12]^	8696333
rs1799945		HFE protein can enhance the iron transport regulator hepciden by binding to TFR2, thereby inhibiting the intestinal enterocyte and macrophage iron export protein ferroportin.^[13]^	19254567
rs855791	TMPRSS6	TMPRSS6 increases iron uptake by inhibiting hepciden production during systemic iron depletion.^[14]^	25550162

SNP = Single-nucleotide polymorphisms, HFE = hemochromatosis, TMPRSS6 = transmembrane protease serine 6 gene, TRF = transferrin receptor.

### 2.2. Outcome data sources

Association estimates between the SNPs and risk of lung cancer were derived from a GWAS summary performed by the International Lung Cancer Consortium (ILCCO),^[10]^ which conducted a meta-analysis of 4 lung cancer GWAS, including the MD Anderson Cancer Center GWAS, the Institute of Cancer Research GWAS, the National Cancer Institute GWAS, and the International Agency for Research on Cancer GWAS. These lung cancer databases contained information for 11,348 individuals of European ancestry with lung cancer (cases) and 15,861 peers (controls).

### 2.3. Selection of instrumental variables

The selected instrumental variables in our MR study met 3 criteria as follows: (1) instrumental variables were robustly correlated with the exposure (iron status); (2) instrumental variables had influenced the outcome (lung cancer) only via the exposure (iron status); and (3) instrumental variables were not associated with confounders in the relationship between exposure (iron status) and outcome (lung cancer).^[15]^ F-statistic is a common method for evaluating instrument strength, and only SNPs with an F-statistic > 10 were used in our study to avoid potential weak instrumental bias.^[16]^ Because the linkage disequilibrium between the 2 loci (rs1800562 and rs1799945) within the HFE gene was low (r^2^ < 0.01), the 3 SNPs associated with all 4 iron biomarkers (rs1800562, rs1799945, and rs855791) were considered as candidate instrumental variables in the mendelian randomization main analysis.

## 3. Statistical analysis

The 2-sample MR was performed for testing the causal relationship between iron status and lung cancer. The SNPs (rs1800562, rs1799945, and rs855791) assigned with the same effect allele were first matched in different data sources, and then the association of each SNP with lung cancer was weighted by its association with iron status. MR estimates were combined using the inverse-variance-weighted (IVW) method, which provided accurate estimates when all SNPs met the criteria for being valid variance variables.^[17]^ The weighted median and MR-Egger regression methods were performed as complementary analyses. The weighted median can provide consistent estimates when more than 50% of information for the analysis comes from valid instruments. The MR-Egger technique detected and adjusted for pleiotropy (*P* for intercept < 0.05), which assumed estimates of low precision,^[18,19]^ and used multiple analysis methods to verify stability of results. Odds ratios (ORs) of lung cancer were scaled to 1 standard deviation (SD) increment of genetically predicted serum iron, log_10_ ferritin, transferrin saturation, and transferrin levels in all analyses. The power was calculated by the online tool named mRnd (https://shiny.cnsgenomics.com/mRnd/),^[20]^ and the results are displayed in Supplementary Table 1, http://links.lww.com/MD/G902. Following Bonferroni correction, associations with p-values below 0.013 (where *P* = .05/4 exposures) were regarded statistically significant.

## 4. Sensitivity analysis

According to the criteria that instrumental variables should influence the outcome only via the exposure (iron status in the present study),^[21]^ the presence of horizontal pleiotropy would violate this assumption; therefore, more robust statistical sensitivity analyses were needed to verify the validity of the causal inference. Statistical sensitivity analyses normally require more than 3 instruments, thus we used SNPs affiliated with at least one of the iron markers (5 SNPs for serum iron, 8 SNPs for transferrin, 6 SNPs for ferritin, and 5 SNPs for transferrin saturation’ Table 1) in the sensitivity analysis. MR-Egger regression was used to determine the intercept and *P*-value to test the directional horizontal pleiotropy. Heterogeneity tests were conducted by performing Cochran Q test of IVW and MR-Egger (*P* < .05).^[17,22]^ The third assumption of MR analysis was that the instrumental variables should not be associated with any confounders correlated with both iron status and lung cancer, otherwise the causal effect would not be accurately estimated. A PhenoScanner V2 database was used to find other phenotypes related to the selected SNPs^[23]^ and were verified as to whether the result of MR analysis was robust or not by manually removing the SNPs. All statistical analyses were 2-sided, and all MR analyses were performed in R (version 4.1.0) using the package “TwoSampleMR” (version 0.5.5).^[24]^

## 5. Results

Three SNPs associated with all 4 iron biomarkers (rs1800562, rs1799945, and rs855791) were used as instrumental variables in the mendelian randomization main analysis. We observed a 3.8%, 0.7%, 7.4%, and 3.3% variance for serum iron, ferritin, transferrin, and transferrin saturation levels, respectively. F statistics of SNPs used in the mendelian randomization main analysis ranged from 47 to 2127 showed that all SNPs were strong instrumental variables (Supplementary Table 1, http://links.lww.com/MD/G902).

**Figure 2.**
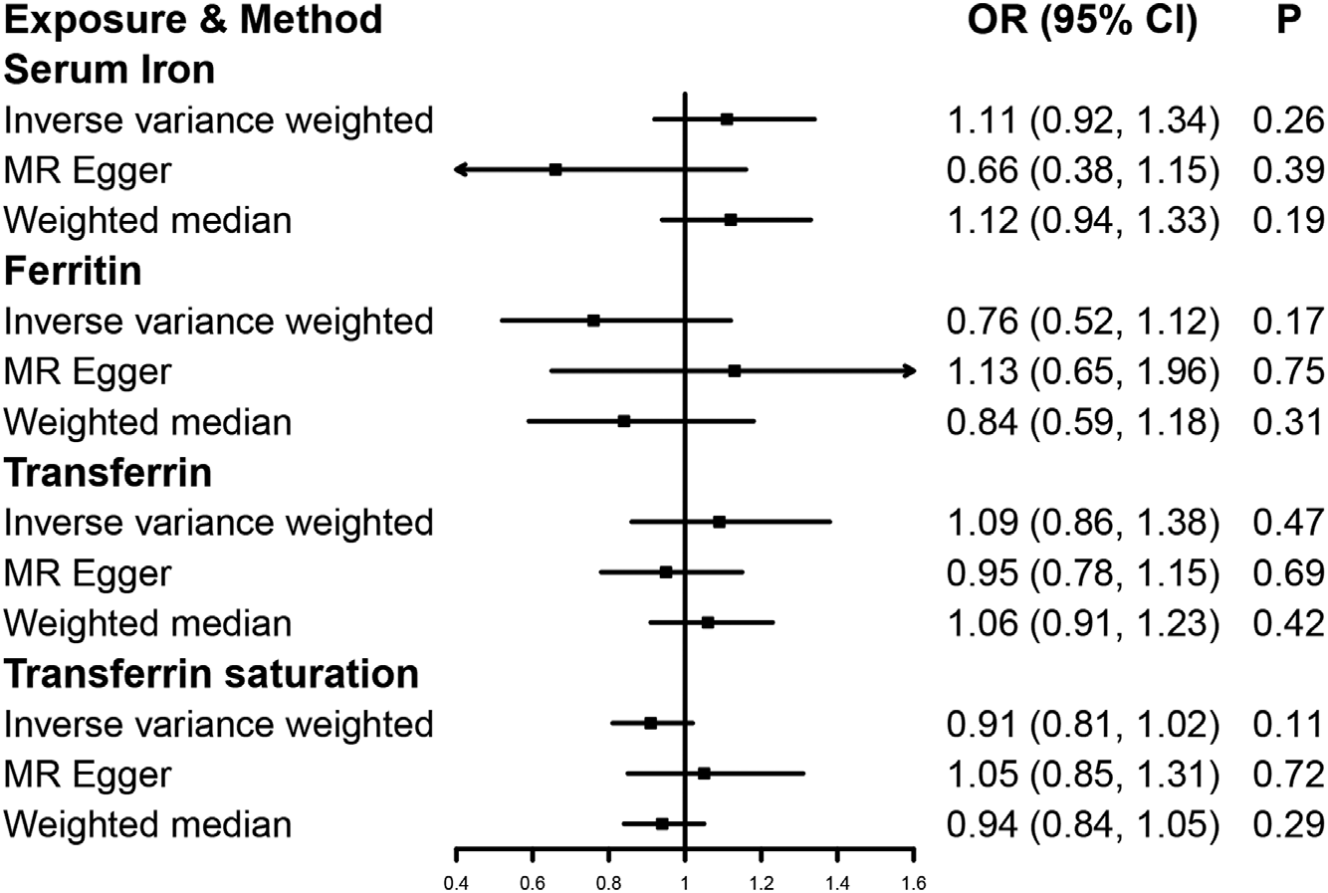
Mendelian randomization estimates the association of genetically-predicted iron status and the risk of lung cancer. CI, indicates confidence interval; OR, odds ratio; SD, standard deviation.

The results of the MR analysis indicated there was no association of high iron status with lung cancer risk. The ORs were 1.11 [95% confidence interval (CI), 0.92, 1.34; *P* = .26], 0.76 (95% CI, 0.52, 1.12; *P* = .17), 1.09 (95% CI, 0.86, 1.38; *P* = .47), and 0.91 (95% CI, 0.81, 1.02; *P* = .11) for 1 SD increase in serum iron, ferritin, transferrin, and transferrin saturation in IVW, respectively (Fig. 2). The results of the supplementary analysis and sensitivity analysis were both consistent with those of IVW (Figure 2 and Supplementary Figure 1, http://links.lww.com/MD/G902). There was no observed indication of heterogeneity (*P* for Q > 0.05) or pleiotropy (*P* for intercept > 0.05 in the MR-Egger analysis) in the sensitivity analysis (Supplementary Table 2, http://links.lww.com/MD/G902). Using the PhenoScanner V2 database, the SNPs (rs1800562, rs1799945, rs855791) used in the MR main analysis were associated with blood cells, lipoprotein, blood pressure, and other traits at genome-wide significance (Supplementary Table 3, http://links.lww.com/MD/G902). Additionally, rs1800562 was related to low-density lipoprotein (LDL). A prospective study published in 2019 proved the correlation between low-density lipoprotein and risk of lung cancer.^[25]^ Nevertheless, removing rs1800562 in the MR analysis failed to change the association between iron status and the risk of lung cancer (Supplementary Table 4, http://links.lww.com/MD/G902, all *P* > .013).

## 6. Discussion

We found that iron status was not causally related to the risk of lung cancer and that causal estimation results of all considered iron status biomarkers were similar. Small differences in estimates of causal effects and confidence interval widths for each marker can be explained by chance and possibly differential measurement error across markers, rather than indicating distinct causal pathways. In our main analysis, we included 3 SNPs that were significantly associated with all iron status markers and had consistent effects on systemic iron status to minimize the risk of invalid instruments. However, only using 3 SNPs did not have the additional power that might be afforded with considering all genetic variants associated with any iron status marker at genome-wide significance. Therefore, in the sensitivity analysis, we used all genetic variants associated with each iron status marker and obtained results consistent with the main analysis, although there were minor differences in the estimates of causal effects and confidence interval widths. To avoid the influence of pleiotropy on MR estimation,^[26]^ we removed rs1800562 associated with LDL from the main analysis and the results of the MR estimates did not substantially change, suggesting that our results were unlikely to be influenced by lipid levels. In addition, no bias was detected in the MR-Egger method in the pleiotropy test. In summary, the overall analysis and conclusions of our study do not appear to be significantly biased, and to our knowledge, this study was the first to use MR to examine the relationship between iron status and lung cancer.

The carcinogenicity of iron has been clearly demonstrated in animal models and human studies,^[27]^ and several mechanisms could explain the potential correlation between iron status and lung cancer. First, iron can produce DNA-damaging oxygen radicals and trigger cancer-causing mutations.^[28]^ Second, previous studies have shown that a large amount of redox-active iron was concentrated in epithelial lining fluid of the lung normal.^[29]^ In addition, heme iron can induce the formation of endogenous N-nitroso compounds (NOC), which can act as tissue-specific carcinogens.^[30]^ The results of many observational studies have shown a potential correlation between iron status and lung cancer risk. A case-control study involving 923 cases found a positive association between iron status and lung cancer risk (OR, 1.45; 95%CI, 1.03–2.06).^[4]^ Similar results were shown in another retrospective study called the Iowa Women’s Health Study (RR, 8.97; 95% CI, 1.29–62.51).^[31]^ However, the opposite conclusion has been observed in some previous studies. NIH-AARP Diet and Health Research showed that the higher the dietary iron intake, the lower the risk of lung cancer.^[32]^ A case-control study of 1139 patients found that high iron intake reduced lung cancer by 19 to 34%.^[33]^ A prospective study that evaluated the association of serum ferritin, iron, transferrin concentrations, and transferrin saturation with cancer risk found no significant association between these iron status markers and the risk of colorectal, prostate, or lung cancers.^[5]^ In addition, a meta-analysis of iron status and lung cancer risk found no significant correlation,^[34]^ which was consistent with the results of our study. Several factors may explain the discrepancy between the results that have been reported in epidemiological studies on iron status and lung cancer risk. First, the difference can be attributed to the diversity of sources of iron intake, which have different effects on lung cancer risk. nonheme iron intake has been associated with a reduced risk of lung cancer,^[35,36]^ whereas red meat has been associated with an increased risk.^[37]^ Second, confounding factors, such as zinc and vitamin C intake and smoking history, cannot be controlled. The MR method based on the random assignment of SNPs used in the present study was able to effectively avoid the confounding bias common in observational studies.

Our study had several advantages. First, the MR study design effectively reduced confounding factors and reversed causation. Second, data were obtained from the Iron State Genetics (GIS) Consortium and the International Lung Cancer Consortium (ILCCO), thus the large sample size made our findings reliable.^[9]^ Finally, GWAS data on both exposure and outcomes were obtained from the European cohort, thereby reduced any ancestry-related bias. The main limitation of the present study was the possibility of horizontal pleiotropy that could not be completely excluded, even though we used a PhenoScanner V2 database to locate other phenotypes related to the selected SNPs and no signs of unbalanced pleiotropy were shown in the heterogeneity test or the MR-Egger analysis. Second, our data sources were mainly from European populations, which may limit the generalization of our findings to other regional populations. Third, the lack of personal information in the publicly available GWAS database made it difficult to perform stratified analysis based on factors, such as age or gender.

## 7. Conclusion

In conclusion, genetically predicted iron status was not causally associated with lung cancer risk; the causal relationship between iron status and lung cancer needs to be further elucidated by additional studies that strictly control for confounding factors.

## Author contributions

Hong Qin and Weibiao Zeng analyzed data and wrote the manuscripts. Yongfu Lou conceived, designed, and supervised the project.

## Acknowledgments

We are extremely grateful to the Genetics of Iron Status Consortium and International Lung Cancer Consortium and all the participants in our study.

## Supplementary Material


