# Supplementary material for: Mendelian randomization study indicates lack of causal associations between iron status and lung cancer
Source: Medicine (Baltimore). 2022 Jul 22;101(29):e29879. doi: 10.1097/MD.0000000000029879 (PMC9302260; doi:10.1097/MD.0000000000029879)
Supplement: Supplementary file 1 [file medi-101-e29879-s001.pdf]

Supplementary Figure 1. Associations between genetically-predicted iron status and lung cancer risk using all SNPs in sensitivity analysis.

Cl, indicates confidence interval; OR, odds ratio

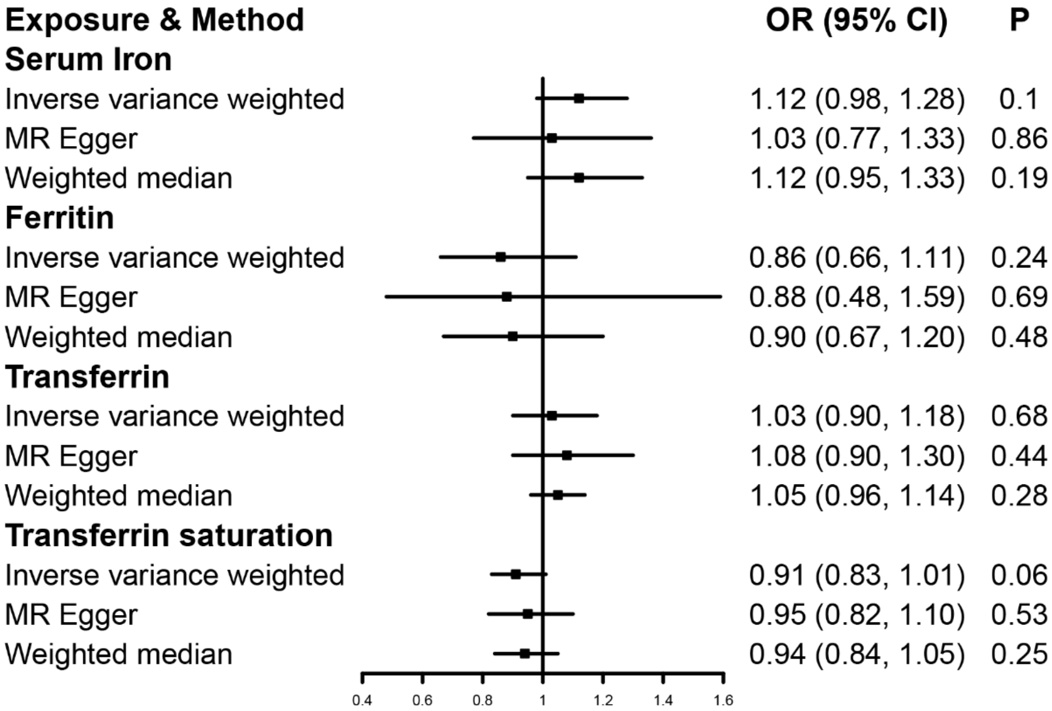

Supplementary Table 1. F statistics for SNPs associated with iron status obtained from the Genetics of Iron Status Consortium GWAS meta-analysis used in the mendelian randomization main analysis.

	**Iron**		**Transferrin Saturation**		**Log_{10} Ferritin**		**Transferrin**	
SNPs	R2 (%)	F	R2 (%)	F	R2 (%)	F	R2 (%)	F
rs1800562	1.3	668	4.2	2127	0.5	256	2.9	1446
rs1799945	0.9	450	1.4	676	0.1	53	0.3	163
rs855791	1.6	806	1.8	889	0.1	73	0.1	47

SNP, indicates single nucleotide polymorphism; F, F statistic; R^2, percentage of the iron marker variation explained by the SNP.

Supplementary Table 2. Heterogeneity test and pleiotropy test following sensitivity analysis using all SNPs associated iron status.

Exposure	Method	Q	Q_df	Q_pval	intercept	intercept_pval
Serum iron	MR Egger	3.96	3	0.27	0.01	0.55
	Inverse variance weighted	4.56	4	0.34	NA	NA
Ferritin	MR Egger	5.94	4	0.2	0	0.93
	Inverse variance weighted	5.95	5	0.31	NA	NA
Transferrin	MR Egger	18.25	6	0	0	0.47
	Inverse variance weighted	20.07	7	0	NA	NA
Transferrin saturation	MR Egger	2.75	3	0.43	0	0.51
	Inverse variance weighted	3.29	4	0.51	NA	NA

df, degrees of freedom; pval, p value; NA, not applicable.

Supplementary Table 3. Associations of the three SNPs used in the MR main analysis with other traits at the genome-wide significance level.

SNP	Chr	Gene	EA	Trait	Beta	p value	Trait	Beta	p value
rs1800562	6	HFE	A	Mean corpuscular hemoglobin	0.3	0	HbA1c	-0.04	4.7×10^{-28}
				Red cell distribution width	-0.192	7.7×10^{-200}	Diastolic blood pressure	0.394	9.0×10^{-17}
				Reticulocyte count	0.109	1.1×10^{-63}	Low density lipoprotein	-0.062	8.3×10^{-14}
				Erythrocyte indices	NA	1.0×10^{-46}	Height	0.022	6.3×10^{-12}
				Pulse rate	0.031	6.8×10^{-11}			
rs1799945	6	HFE	G	Mean corpuscular hemoglobin	0.193	0	HbA1c	-0.021	3.7×10^{-19}
				Red cell distribution width	-0.133	2.8×10^{-161}	Blood pressure	NA	2.0×10^{-15}
				Reticulocyte count	0.057	2.0×10^{-30}	Platelet count	-0.035	5.0×10^{-12}
				Hypertension	NA	2.0×10^{-10}			
rs855791	22	TMPRSS6	G	Mean corpuscular hemoglobin	0.17	0	HbA1c	-0.017	3.4×10^{-28}
				Red cell distribution width	0.125	2.8×10^{-271}	Platelet count	-0.032	2.2×10^{-18}
				Reticulocyte count	0.042	8.2×10^{-31}			

SNP, single nucleotide polymorphism; MR, Mendelian randomization; Chr, indicates chromosome; EA, effect allele.

PhenoScanner V2 link is as follows: http://www.phenoscanner.medschl.cam.ac.uk

Related or repeated traits were counted once.

Supplementary Table 4. Association estimates of iron status and the risk of lung cancer after excluding rs1800562 using the inverse variance weighted analysis.

Exposure	Beta	SE	OR (95% Cl)	p value*
Serum Iron	0.19	0.08	1.21 (1.03, 1.42)	0.019
Ferritin	-0.58	0.28	0.56 (0.32, 0.96)	0.037
Transferrin	0.34	0.36	1.40 (0.69, 2.81)	0.346
Transferrin saturation	-0.16	0.08	0.85 (0.72, 1.00)	0.044

SE, standard error; OR: odds ratio; 95% CI, 95% confidence interval

* p values below 0.013 (where p = 0.05/4 exposures) were regarded as statistically significant.
